# Green synthesized apigenin conjugated gold nanoparticles inhibit cholangiocarcinoma cell activity and endothelial cell angiogenesis *in vitro*

**DOI:** 10.1016/j.heliyon.2022.e12028

**Published:** 2022-12-01

**Authors:** Nipaporn Ngernyuang, Molin Wongwattanakul, Wannit Charusirisawad, Rong Shao, Temduang Limpaiboon

**Affiliations:** a Chulabhorn International College of Medicine, Thammasat University, Pathum Thani 12120, Thailand; b Centre for Research and Development of Medical Diagnostic Laboratories, Faculty of Associated Medical Science, Khon Kaen University, Khon Kaen 40002, Thailand; c Development of Pharmacology, Shanghai Jiao Tong University School of Medicine, Shanghai 200025, China

**Keywords:** Apigenin, Gold nanoparticles, Green synthesis, Cholangiocarcinoma, Apoptosis

## Abstract

Cholangiocarcinoma (CCA) is a rare malignancy of the biliary tract with extremely poor clinical outcomes due to a lack of effective therapies to improve disease management. The emerging green synthesis of gold nanoparticles (AuNPs) has extensively provided their use in biomedical applications. In this study, we developed AuNPs via reducing gold salts with apigenin (4′,5,7-trihydroxyflavone). The synthesized apigenin-conjugated AuNPs (api-AuNPs) were physicochemically characterized by various techniques before evaluation their biological and functional inhibition in a CCA cell line, KKU-M055. The mean size of api-AuNPs was 90.34 ± 22.82 nm with zeta potential of -36 ± 0.55. The half-maximal inhibitory concentration (IC_50_, 0.8 mg/mL) of api-AuNPs on cell proliferation of KKU-M055 was 1.9-fold less than that of an immortalized human cholangiocyte cell line, MMNK1 (IC_50_, 1.5 mg/mL). Moreover, api-AuNPs induced cell apoptosis via the up-regulation of *Bax*, *Bid*, and *Caspase 3*, and down-regulation of *Bcl2*, leading to elevated caspase 3/7, 8, 9 activities and reactive oxygen species (ROS) production. The api-AuNPs significantly inhibited the migration of KKU-M055 cells and suppressed the proliferation, migration, and *in vitro* tube formation of vascular endothelial cells. Collectively, our findings indicate the dual abilities of api-AuNPs that potentially inhibit cancer cell growth and motility as well as endothelial cell-mediated angiogenesis, which may offer a novel therapeutic avenue to treat CCA patients effectively.

## Graphical abstract

**Figure:**
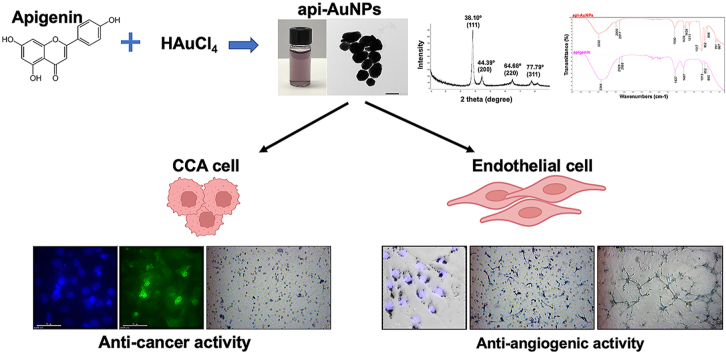

## 1 Introduction

Cholangiocarcinoma (CCA) is the second most common type of primary hepatic malignancy. The incidence of CCA is continuously increasing worldwide and the highest incidence of CCA in Thailand has been noticed in the northeastern region [1]. The current standard treatment for advanced CCA is chemotherapy with gemcitabine-based doublets. However, increasingly and rapidly unexpected chemoresistance has emerged as the primary challenge, resulting in poor prognosis and sustaining high mortality [2]. In addition, the malignant transformation of cancer is intimately associated with the microenvironment alteration that involves vascular angiogenesis and immune cell dysfunction. Accumulating evidence has indicated that increased vascularization of CCA is strongly correlated with poor prognosis [3]. While the molecular mechanisms underlying tumor angiogenesis have been notably deciphered, little is known regarding the effective inhibition of tumor angiogenesis in the development of CCA [4]. Therefore, additional effort is urgently needed to develop novel agents to improve therapeutic strategies for both anti-cancer and anti-angiogenic effects in this disease.

Over the past few years, the applications of nanotechnology exhibit significant potential in the fields of medicine and healthcare, including in molecular imaging, diagnostic tools, drug delivery systems, tissue-engineered constructs, and pharmaceutical therapeutics [5]. Nanoparticles (NPs) are of great nanomaterials due to their extremely small size (<100 nm) and large surface-to-volume ratio. Gold nanoparticles (AuNPs), which typically show high stability with respect to other metal nanoparticles, have been proposed for different diagnostic and therapeutic applications [6, 7] such as cancer hyperthermia [8, 9], cancer radiation therapy [10, 11], cancer drug delivery [12], antimicrobials [13, 14], and biosensors [15, 16]. AuNPs are commonly developed through various methods including conventional, chemical, and physical synthesis. Given material safety and complexity in the generation of AuNPs that routinely employs toxic chemicals or requires high energy, a novel method utilizing natural materials has been developed to yield green AuNPs. More interestingly, these biosynthesized AuNPs have been found to have the ability to function as antimicrobial, antiviral, anti-inflammatory, antioxidant, and anticancer activity [17, 18, 19, 20, 21, 22, 23].

Apigenin is a flavonoid (4′,5,7-trihydroxyflavone, C_15_H_10_O_5_) compound derived from vegetables and fruits with a molecular weight of 270.24 Dalton [24]. Apigenin has been used to treat some diseases including inflammation [25], viral infections [26, 27], oxidation disorders [28], and cancer [24]. Several lines of research evidence have revealed that apigenin can inhibit cell proliferation and angiogenesis, and induce autophagy and apoptosis in colorectal cancer [29], breast cancer [30], lung cancer [31], cervical cancer [32], ovarian cancer [33], prostate cancer [34], and skin cancer [35]. However, its applications in clinical trials have been largely limited due to its poor water solubility (1.35 μg/mL) and insufficient levels in targeted cells [36]. Thus far, modification of its properties to improve the bioavailability and subsequent therapeutic efficacy has received significant attention. Apigenin-coated AuNPs (ap-AuNPs) have been found to inhibit cancer cell proliferation and promote apoptosis in human epidermoid carcinoma cells [37]. Moreover, ap-AuNPs can enhance the efficiency of radiation therapy *in vitro* treatment of lung cancer cells [38]. In addition, Amini et al reported that *in vitro* photothermal therapy treatment with ap-AuNPs leads to the death of the lung cancer cell and normal fibroblast cell through apoptosis-mediated cell death [39]. However, there has not been any report of the anti-cancer activity of apigenin-mediated AuNPs in CCA.

In this study, we employed apigenin as a reducing agent to induce conjugation with AuNPs (api-AuNPs) that facilitate the delivery efficiency. Multiple high technological techniques were engaged to fully characterize physicochemial properties of the api-AuNPs including the Ultraviolet-visible (UV-vis) spectrum, transmission electron microscopy (TEM), x-ray diffraction (XRD), zeta potential, Fourier transform infrared spectrophotometry (FTIR), and high-performance liquid chromatography (HPLC). The biological effects of api-AuNPs on a CCA cell line, KKU-M055 and human microvascular endothelial cells (HMVECs) were evaluated.

## 2 Experimental section

### 2.1 Synthesis of apigenin conjugated gold nanoparticles (api-AuNPs)

Gold nanoparticles was produced according to the instruction reported in literature with modification [37]. Brieﬂy, a stock solution of apigenin was dissolved in dimethyl sulfoxide (DMSO) (all from Sigma Aldrich, St. Louis, MO, USA). The 5 mM solution of apigenin in DMSO was added to deionized water (DI) water and adjust the pH to 10 with 150 mM of K_2_CO_3_. Then, 10 mM of chloroauric acid trihydrate (HAuCl_4_·3H_2_O, ≥49.0% Au basis) (Sigma-Aldrich) was added dropwise while mixing the solution of apigenin at room temperature, generating final concentration of apigenin and HAuCl_4_ was 0.3 mM and 1.5 mM, respectively. The api-AuNPs solution was stirred for 48 h at room temperature and collected by centrifugation at 12,000 rpm for 10 min at 10 °C. The pellet was dispersed with DI and washed twice to remove unreacted compounds. For cell culture experiments, the api-AuNPs pellets were dispersed in cell culture medium.

### 2.2 Physicochemical characterization of api-AuNPs

The api-AuNPs were characterized by various analytical techniques. The reduction of gold ions was monitored by recording the UV-vis spectrum in the wavelength range 400–900 nm using a Thermo Varioskan Flash Multi Detection microplate reader (Thermo Fisher Scientific, Tewksbury, MA, USA). The morphology and size of the api-AuNPs were determined using a TEM (JEM-2100 Plus; JEOL, Tokyo, Japan). The pellet was dispersed in DI water and dropped on a carbon coated copper grid. The high-resolution transmission electron microscopy (HRTEM) image acquired at an accelerating voltage of 200 kV. The stability in zeta potential of api-AuNPs in DI water and api-AuNPs in cell culture medium were measured by ZetaSizer Nano ZS (Malvern Instruments Ltd., Worcs, UK). XRD patterns were evaluated using the D8 ADVANCE Bruker AXS diffractometer (Bruker, Karlsruhe, Germany). The crystalline structure was analyzed at a voltage of 40 kV and a current of 40 mA using Cu radiation (1.5406 Å) in the range of 10–90° in 2θ angles with a scan speed of 0.02°/step. The FTIR was employed to determine the functional groups of apigenin on the surface of api-AuNPs using Nicolet iS50 FTIR spectrometers (Thermo Fisher Scientific). Each spectrum was recorded as the average of 64 scans with 4 cm^−1^ resolutions in the wavenumber range of 400–4000 cm^−1^. To confirm the purity of api-AuNPs, the free apigenin molecules in supernatant of each washing step were observed by UV-vis spectrum.

### 2.3 Cell culture

Human intrahepatic CCA cell line KKU-M055 was established in the Cholangiocarcinoma Research Institute, Faculty of Medicine, Khon Kaen University. An immortalized human cholangiocyte cell line MMNK1 was used as the reference cholangiocyte. KKU-M055 and MMNK1 cells were cultured in a dulbecco's modified eagle medium (DMEM) supplemented with 1% penicillin/streptomycin (P/S) and 10% fetal bovine serum (FBS) (all from Life Technologies, Paisley, UK). HMVECs were cultured in endothelial basal medium (EBM-2) supplemented with 10% FBS, 1% P/S, 10 ng/mL human epidermal growth factor (hEGF), and 1 μg/mL hydrocortisone (all from Lonza, Walkersville, MD, USA). All cells were cultured in a humidified incubator at 37 °C with 5% CO_2_.

### 2.4 Cell cytotoxicity

Cell cytotoxicity was assessed by measuring the viable cells using celltiter 96® aqueousone solution cell proliferation assay (3-(4,5-dimethylthiazol-2-yl)-5-(3-carboxymethoxyphenyl)-2-(4-sulfophenyl)-2H-tetrazolium; MTS assay) (Promega, Madison, WI, USA. Briefly, the CCA cells were seeded at a density of 5×10^3^ cells per well of 96-well plates in triplicate and grown for 24 h to reach 80% confluence. The medium was replaced with fresh medium containing different concentrations of api-AuNPs (0.3–1.6 mg/mL) and incubated for 48 h. After treatment, MTS solution was added to the wells and plates were incubated for 1 h. The supernatant was transferred to a new 96-well plate to prevent the interference of AuNPs absorption and determined cellular absorbance at 490 nm using a Thermo Varioskan Flash Multi Detection microplate reader (Thermo Fisher Scientific). The percentage of viable cells was calculated by normalization to the untreated controls. The IC_50_ (concentration causing a 50% inhibition with respect to the controls) value was calculated using a linear regression equation.

### 2.5 Annexin V-FITC apoptosis assay

FITC-annexin V apoptosis detection kit I (BD Biosciences, San Jose, CA, USA) was utilized to evaluate cell death induced by api-AuNPs. Briefly, 2×10^6^ cells in a 6-well plate were incubated with different concentrations of api-AuNPs (0, 0.8, and 1.2 mg/mL) in triplicate for 48 h. Untreated cells were used as the control group. After incubation, cells were collected and washed once with cold phosphate buffered saline (PBS), and resuspended in 1X binding buffer at a density of 1×10^6^ cells/mL. Then, the cells were incubated with FITC-annexin V and propidium iodide (PI) for 15 min at room temperature in the dark. The stained cells were detected by Becton Dickinson flow cytometer (BD Biosciences). The data were analyzed by BD FACSDiva software (BD Biosciences).

### 2.6 Apoptotic cell staining

Apoptosis was assessed by nuclear morphology after treatment with the fluorescent DNA-binding dye, Hoechst 33258 solution (5 μg/mL) (Sigma-Aldrich). Briefly, 5×10^5^ cells were seeded into a 24-well plate and treated with different concentrations of api-AuNPs (0, 0.8, and 1.2 mg/mL) in triplicate for 48 h. After treatment, the cells were fixed by 100% cold methanol for 10 min and washed twice with cold PBS followed by incubation with Hoechst 33258 solution for 10 min at room temperature. The morphologic changes in apoptotic nuclei were observed and photographed under the fluorescence microscope. The mitochondrial transmembrane potential assay was assessed by using Rhodamine 123 (Rho 123 staining) (Sigma-Aldrich). Briefly, the Hoechst 33258-stained cells were incubated with Rho 123 (2.5 μg/mL) for 15 min at room temperature in the dark. After incubation, the cells were washed with PBS and observed under a fluorescence microscope.

### 2.7 Reactive oxygen species (ROS) formation

To evaluate the intracellular ROS production, a non-fluorescent probe 2′,7′-Dichlorofluorescin diacetate (DCFH-DA; Sigma-Aldrich) assay was performed. Briefly, 5×10^3^ cells were seeded per well in 96-well plates and treated with different concentrations of api-AuNPs (0, 0.8, and 1.2 mg/mL) in triplicate for 24 h. Then, the cells were washed with PBS and stained with DCFH-DA at room temperature for 30 min. Fluorescence intensity was measured using a Thermo Varioskan Flash Multi Detection microplate reader (Thermo Fisher Scientific) with an excitation at 485 nm and emission at 537 nm, respectively. Data are presented as the mean percentage of ROS production compared to untreated cells set as 100% ROS.

### 2.8 Caspases activity assay

Caspase-8, 9, and 3/7 activities were detected using a caspase Glo-8, 9, and 3/7 assay kit (Promega). KKU-M055 cells (5×10^3^ cells) were seeded into black 96-well plates in triplicate and treated with or without 0.8 mg/mL of api-AuNPs for 24 and 48 h. After 1 h incubation with caspase reagents, the luminescent signal was measured using a Thermo Varioskan Flash Multi Detection microplate reader (Thermo Fisher Scientific). Data are presented as the percentage change in comparison with the untreated cells, which were arbitrarily assigned as 100% activity.

### 2.9 Reverse transcription - polymerase chain reaction (RT-PCR)

Total RNA was extracted from api-AuNPs-treated and untreated KKU-M055 cells using GF-1 total RNA extraction kit (Vivantis Technologies, Selangor, Malaysia) according to the manufacturer's instructions. The first strand complementary DNA (cDNA) was synthesized by reverse transcription with oligo d(T) primers using the Improm II™ reverse transcriptase system (Promega). RT-PCR was performed to detect the expression level of apoptotic genes (*Bax*, *Bid*, and *Caspase 3*), pro-survival gene (*Bcl-2*) and reference gene (*Actin*), using a Toptaq Master Mix Kit (Qiagen, Hilden, Germany). The PCR reaction of the above genes was carried out in a T100 Thermal Cycler PCR system (Bio-Rad Laboratories, Hercules, CA, USA) using the primer sequences as shown in Table 1.

**Table 1:** Primer sequences for RT-PCR.

Genes	Forward primer sequences (5′–3′)	Reverse primer sequences (5′–3′)	Product size (bp)
*Bax*	CATCCAGGATCGAGCAGG	CATGTCAGCTGCCACTCGG	208
*Bid*	CAAGAAGGTGGCCAGTCACAC	GCTCCGTCTACTCTGGAAGC	199
*Caspase 3*	GTTGATGATGACATGGCGTG	GTTGCCACCTTTCGGTTAA	203
*Bcl-2*	GACTTCGCCGAGATGTCCAG	GTTCAGGTACTCAGTCATCCA	216
*Actin*	CTCCTCCCTGGAGAAGAGCT	GCAATGCCAGGGTACATGGT	231

### 2.10 Cell migration assay

The effect of api-AuNPs on cell migration was performed in a Transwell system (Costar Corning, Kennebunk, ME, USA). Briefly, KKU-M055 cells or HMVECs were treated with different concentrations of api-AuNPs (0, 0.8, and 1.2 mg/mL) for 48 h. Untreated cells were used as the control group. After treatment, 2×10^5^ cells were suspended in serum-free medium and seeded into the upper chamber in triplicate, while medium containing 10% FBS was added to the lower chamber of the Transwell. After 24 h incubation, non-migrating cells residing at the upper side of the filter were gently removed using a cotton swab applicator. Migrating cells attached to the underside of the filter were fixed with 100% methanol for 10 min and stained with 2.5 % crystal violet in 2% ethanol at room temperature for 30 min. The number of migrating cells was counted in six random fields under a light microscope at 200X magnification.

### 2.11 In vitro tube formation assay

The capillary-like structures formed by HMVECs on Matrigel (BD Biosciences) was evaluated. Briefly, HMVECs were treated with different concentrations of api-AuNPs (0, 0.8, and 1.2 mg/mL) in EBM-2 medium containing 10% FBS for 48 h. Untreated cells were used as the control group. The Matrigel was added to each well of a 96-well plate and allowed to polymerize at 37 °C for at least 30 min before the assay. Subsequently, HMVECs (1×10^4^ cells) in the serum-free EBM-2 medium was seeded on a 96-well plate coated with Matrigel in triplicate. After 24 h incubation, the number of tube-like structure formation of each group was quantified in six random fields under a light microscope at 200X magnification.

### 2.12 Statistical analysis

SPSS version 20 software (IBM Corp., Armonk, NY, USA) was used for statistical analyses. Values are presented as the mean ± standard deviation (SD) derived from three independent experiments. Comparisons between the groups were performed with the two-tailed Student's *t*-test. A value of *p* < 0.05 was considered statistically significant.

## 3 Results and discussion

### 3.1 Synthesis and characterization of api-AuNPs

**Figure 1:**
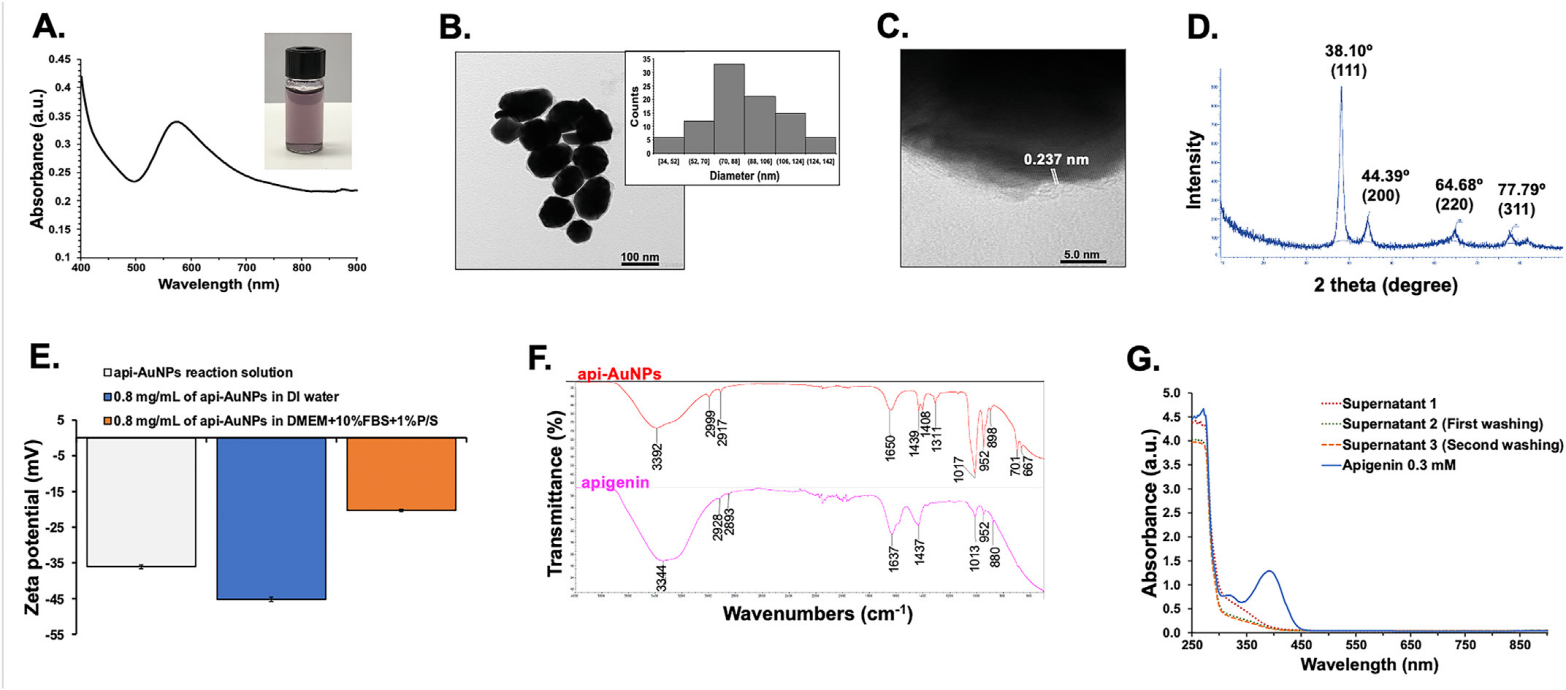
Physicochemical characterization of api-AuNPs. (A) UV-vis spectrum (B) TEM image (left) with different sizes of api-AuNPs (right) (C) HRTEM image (D) XRD pattern (E) Zeta potential value (F) FTIR spectra (G) UV-vis spectroscopic analysis of apigenin (0.3 mM) and supernatant of each washing step.

Gold nanoparticles were synthesized by reducing the gold salt with apigenin. We investigated the bio-reduction of AuNPs using apigenin as the reducing and stabilizing agent at room temperature. The generation of api-AuNPs solution was visually verified by a color change from pale yellow to purple, which indicated the reaction occurrence between Au^3+^ and apigenin. The visible absorption spectrum of colloidal api-AuNPs showed a sharp absorbance peak at approximately λ_max_ = 568 nm (Figure 1A). To analyze the physical properties of the api-AuNPs, we selected 100 gold colloid particles and evaluated the morphology and distribution under TEM. Api-AuNPs displayed the multi-faceted morphology with an average diameter of 90.34 ± 22.82 nm (Figure 1B). In addition, HRTEM defined equally spaced lattice fringes on api-AuNPs (Figure 1C). The lattice fringe separation of api-AuNPs was 0.237 nm, which corresponds to the d-spacing of the (111) plane of gold [40]. The lattice fringe in the HRTEM image was confirmed by XRD that exhibited four distinct diffraction peaks at approximately 38.10°, 44.39°, 64.68°, and 77.79°. Each peak accounted for reflections from the (111), (200), (220), and (311) planes of the face-centered cubic (FCC) Au, respectively (Figure 1D). These peaks were consistent with standard database of the JCPDS card No 04-0784, indicating that the biogenic AuNPs synthesized by apigenin are composed of gold (Au^0^) [41].

The stability of api-AuNPs was subsequently evaluated by a zeta potentiometer. The potential value at either higher than +30 mV or lower than -30 mV indicates the capability of a basically stable suspension, whereby the larger the absolute value, the higher stable potential [42]. The zeta potential value of api-AuNPs was -36 ± 0.55 mV, indicating the stability of the colloidal dispersion of api-AuNPs (Figure 1E). The interaction of AuNPs with biological materials in cell culture medium can cause the aggregation of nanoparticles [43, 44]. Therefore, the stability of api-AuNPs after 48 h incubation in DI water and cell culture medium (DMEM supplemented with 10% FBS and 1% P/S) was also studied using zeta potential measurement. As shown in Figure 1E, the zeta potential of 0.8 mg/mL of api-AuNPs in DI water was -45.20 ± 0.66 mV, while 0.8 mg/mL of api-AuNPs in cell culture medium was decreased to -20.27 ± 0.31 mV, indicating the potential aggregation of api-AuNPs as a result of the protein absorption on the surface. However, a previous study reported that the larger sized AuNPs or their aggregates in cell culture medium exhibit an increased cell uptake rates and increased toxicity [43].

FTIR analysis was carried out to identify the possible functional groups of biomolecules involved in the reduction of gold ions and stabilization of the synthesized AuNPs from apigenin. The FTIR spectra of both apigenin and api-AuNPs were recorded (Figure 1F). The spectrum of pure apigenin appeared at 3344, 2928, 2893, 1637, 1437, 1013, 952, and 880 cm^−1^, respectively. The strong peak at 3344 cm^−1^ was the O–H stretching vibration [45]. The peaks at 2928 and 2893 cm^−1^ were attributed to C–H stretching vibrations of methyl, methylene, and methoxy groups [46]. The peak located at 1637 cm^−1^ was assigned to the C=O stretching vibration. The band at 1437 cm^−1^ was assigned to the C=C stretching in aromatic compounds. The peaks at 1013, 952, and 880 cm^−1^ corresponded to the stretching vibrations of the C–O stretching carboxylic acid group, C–N aromatic, and C–C–O stretching vibrations, respectively [47]. However, in the api-AuNPs, these peaks were shifted to 3392, 2999, 2917, 1650, 1439, 1408, 1311, 1017, 952, 898, 701, and 667 cm^−1^. These shifted peaks clearly suggested that the involvement of carboxyl and hydroxyl groups in apigenin could account for the reduction, capping, and stabilization processes during api-AuNP synthesis.

The removal of the free apigenin molecules is important process for the purification of api-AuNPs product. The free apigenin molecules in supernatant of each washing step were observed by UV-vis spectrum. The representation of A and B ring of apigenin was observed at 390 and 320 nm, respectively (Figure 1G). After twice washing, no trace of apigenin molecule absorbance was observed in the supernatant by UV-vis spectrum (Figure 1G).

### 3.2 The api-AuNPs inhibit proliferation of human CCA cell line KKU-M055

**Figure 2:**
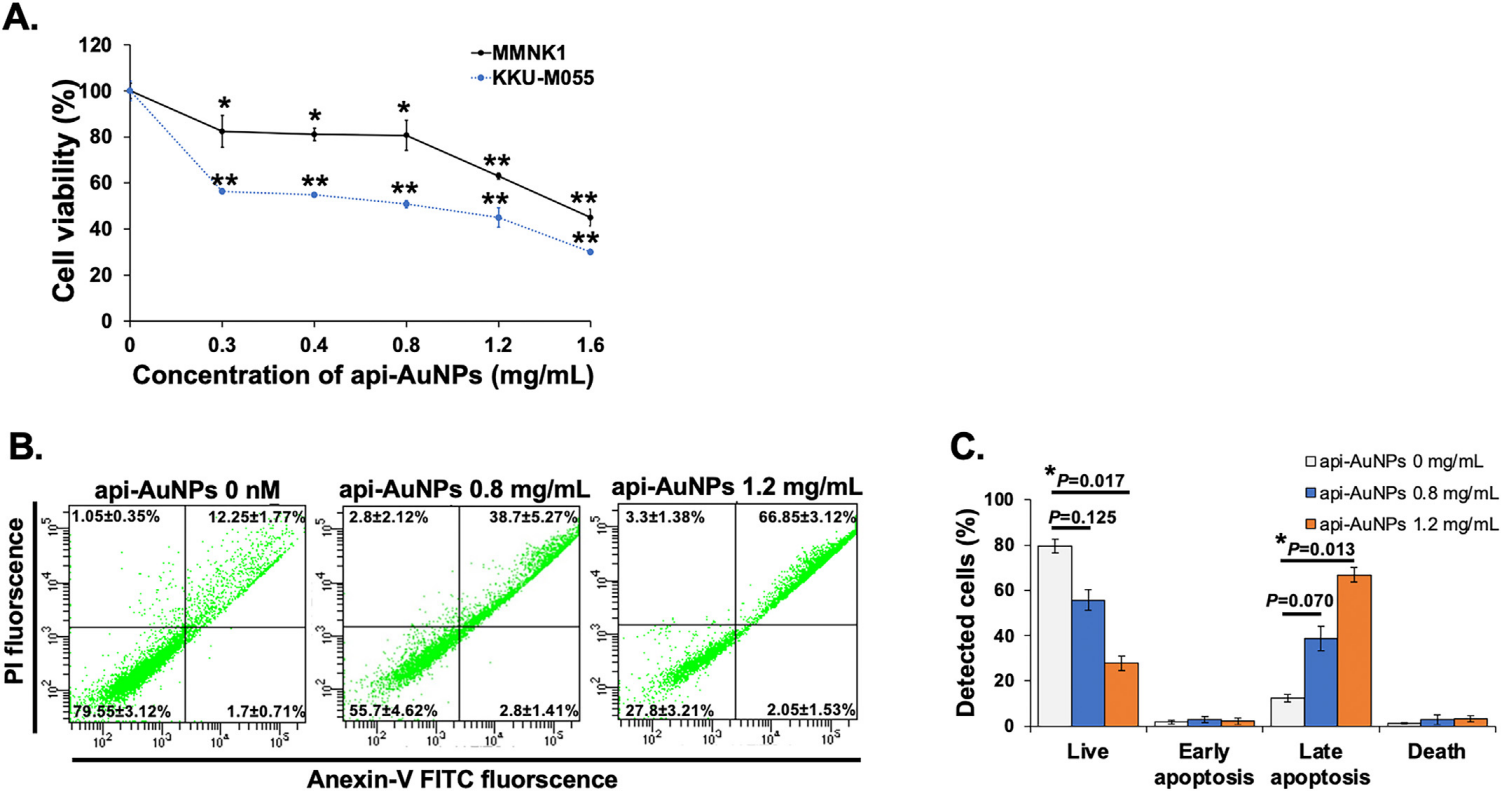
Cell cytotoxicity and flow cytometry analysis. (A) Cytotoxicity effect of api-AuNPs on MMNK1 and KKU-M055 was investigated using MTS assay. (B) Apoptotic population of KKU-M055 was determined by Annexin-V assay. (C) Quantitative data of apoptosis percentages of untreated and treated KKU-M055 cells with different concentrations of api-AuNPs. Values are represented as mean ± SD derived from three independent experiments. Statistically significant differences at: ∗p < 0.05 and ∗∗p < 0.01.

It has been reported that nanoparticles interfere with MTT (3-(4,5-dimethylthiazol-2-yl)-2,5-diphenyltetrazolium bromide) assay [48, 49, 50]. To ascertain that the api-AuNPs did not interfere with MTS, we firstly evaluated MTS assay in the absence and presence of api-AuNPs dispersed in cell culture medium. There was no difference of absorbance peak between the absence and presence of api-AuNPs, indicating that api-AuNPs had no effect on MTS assay. Thus, the MTS assay was performed to investigate the cytotoxic effects of api-AuNPs *in vitro*. An immortalized human cholangiocyte cell line MMNK1 and a human CCA cell line KKU-M055 were treated with various concentrations of api-AuNPs ranging from 0.3 to 1.6 mg/mL for 48 h. Api-AuNPs inhibited the proliferation of KKU-M055 cells in a dose-dependent manner (Figure 2A). The IC_50_ of api-AuNPs on KKU-M055 cells after 48 h treatment was 0.8 mg/mL, 1.9-fold lower than that of MMNK1 cells (1.5 mg/mL), suggesting that api-AuNPs were less toxic to immortalized cholangiocyte cells. The enhanced cytotoxicity of AuNPs against cancer cells is ascribed to the higher uptake of nanoparticles by cancer cells than normal cells, as cancer cells have an atypical metabolism and high proliferation rate relative to normal cells [51, 52]. Similar to our result, Rajendran et al. showed no cytotoxic effect of ap-AuNPs on normal epidermoid cells. However, the IC_50_ of epidermoid squamous carcinoma cells (A431) was 130.3 ± 4.5 μg/mL after 48 h treatment with ap-AuNPs. The cytotoxicity is also dependent on types of target cells, particle size, shape, and surface chemistry of nanoparticles [53]. In addition, phytochemical-coated AuNPs are highly biocompatible with normal cells compared to chemical-coated metal nanoparticles [37, 54, 55].

Based on cytotoxicity result, two different concentrations of api-AuNPs (0.8 mg/mL and 1.2 mg/mL) were selected for further studies. The concentration of api-AuNPs at 0.8 mg/mL was IC_50_ value and 1.2 mg/mL was the IC_75_.

### 3.3 The api-AuNPs induce apoptosis in CCA cells

To address whether the api-AuNPs inhibited apoptosis of KKU-M055 cells, flow cytometry using Annexin V and PI was engaged. Annexin V is a specific phosphatidylserine-binding protein that can be used to detect apoptotic cells, and PI is used to detect necrotic or late apoptotic cells [56]. As shown in Figure 2B and 2C, 80% of live cells and less than 15% of apoptotic KKU-M055 cells were detected in the untreated group. By contrast, 43.62% and 72.20% of apoptotic cells were observed after treatment with 0.8 mg/mL and 1.2 mg/mL of api-AuNPs for 48 h, respectively. Our finding indicated that api-AuNPs induced CCA cell death in a dose-dependent manner.

**Figure 3:**
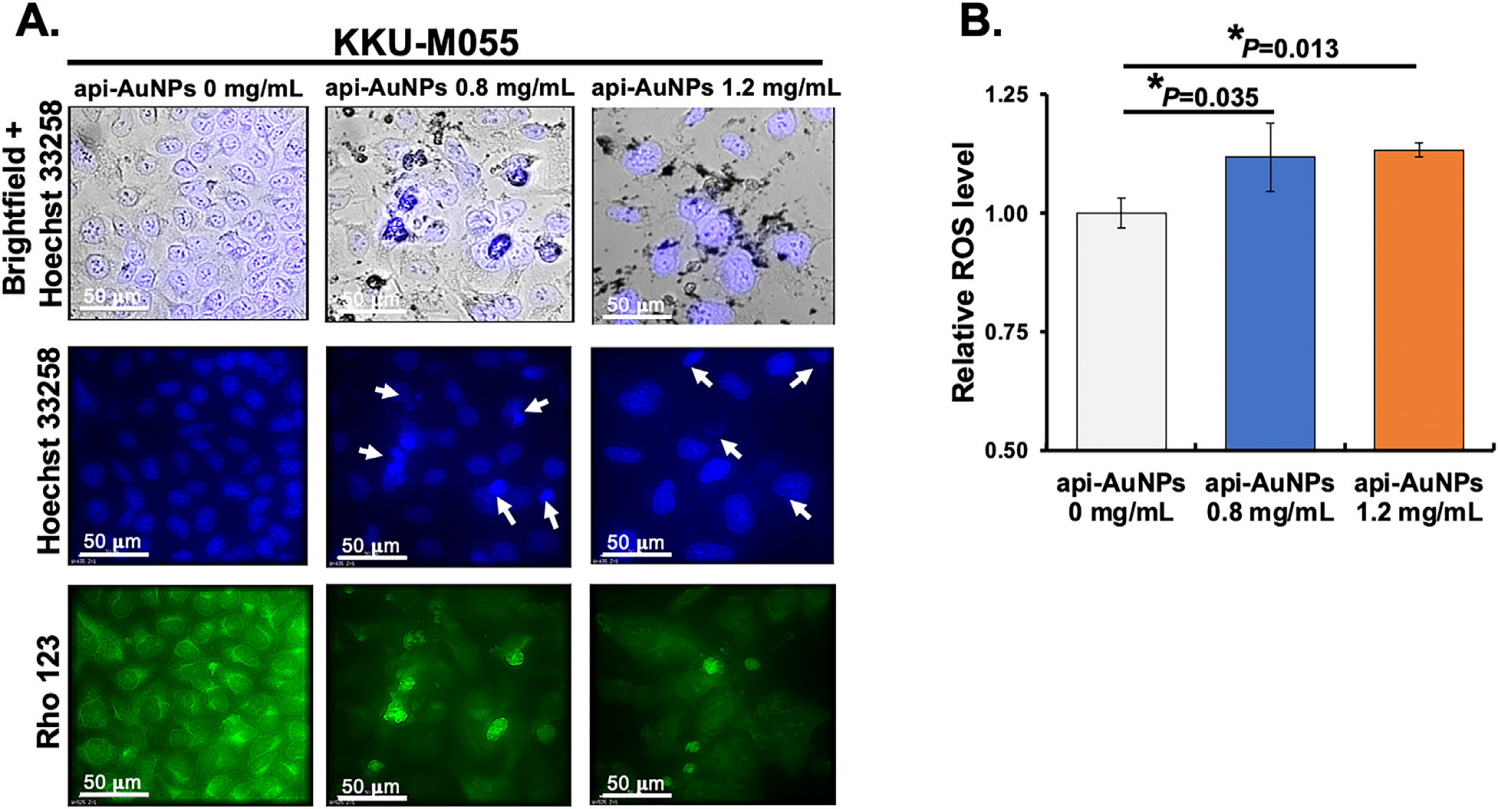
The api-AuNPs induced apoptosis and ROS generation in KKU-M055 cells. (A) Fluorescence micrographs using brightfield + Hoechst 33258 (top row), Hoechst 33258 (middle row), and Rho 123 (bottom row) staining of KKU-M055 cells treated with or without api-AuNPs. The morphological changes are indicated as apoptotic bodies and chromatin condensation (white arrows). (B) DCFH-DA measured ROS levels in terms of relative fluorescence in KKU-M055 cells after api-AuNPs treatment. The results are expressed as the mean ± SD (n = 3).

The apoptotic cells were further confirmed by Hoechst 33258 staining and fluorescence microscopy. The api-AuNPs induced an apoptotic phenotype of KKU-M055 cells compared to untreated cells (Figure 3A, top row). As shown in Figure 3A (middle row), nuclear condensation and fragmentation were observed in the api-AuNP-treated KKU-M055 cells.

To further evaluate whether this apoptosis is associated with loss of mitochondrial membrane potential (ΔΨm), we stained cells with Rho 123 dye that is commonly used to measure the reduction ability of mitochondria to generate a fluorescence product [57]. We found that the api-AuNPs induced a decrease of Rho 123 fluorescence compared to untreated cells (Figure 3A, bottom row), suggesting that api-AuNPs induced mitochondrial damage-associated apoptosis in KKU-M055 cells.

DCFH-DA cell staining was used to evaluate ROS production in the cells. The api-AuNPs significantly increased ROS generation after 24 h treatment (Figure 3B), suggesting that api-AuNP-induced cytotoxicity possibly depended on ROS. In fact, ROS plays a pivotal role in the control of cancer cell growth and death. Increased ROS generation might lead to apoptosis via both extrinsic and intrinsic apoptotic pathways [58]. Our results were consistent with previous studies that the treatment of a number of cancer cells with AuNPs led to cellular ROS accumulation [59, 60]. Moreover, apigenin compounds have been found to inhibit cell viability with an increase of ROS and to cause the loss of ΔΨm, resulting in apoptosis of malignant mesothelioma cell lines [61].

### 3.4 Effect of api-AuNPs on caspase activity in CCA cell lines

**Figure 4:**
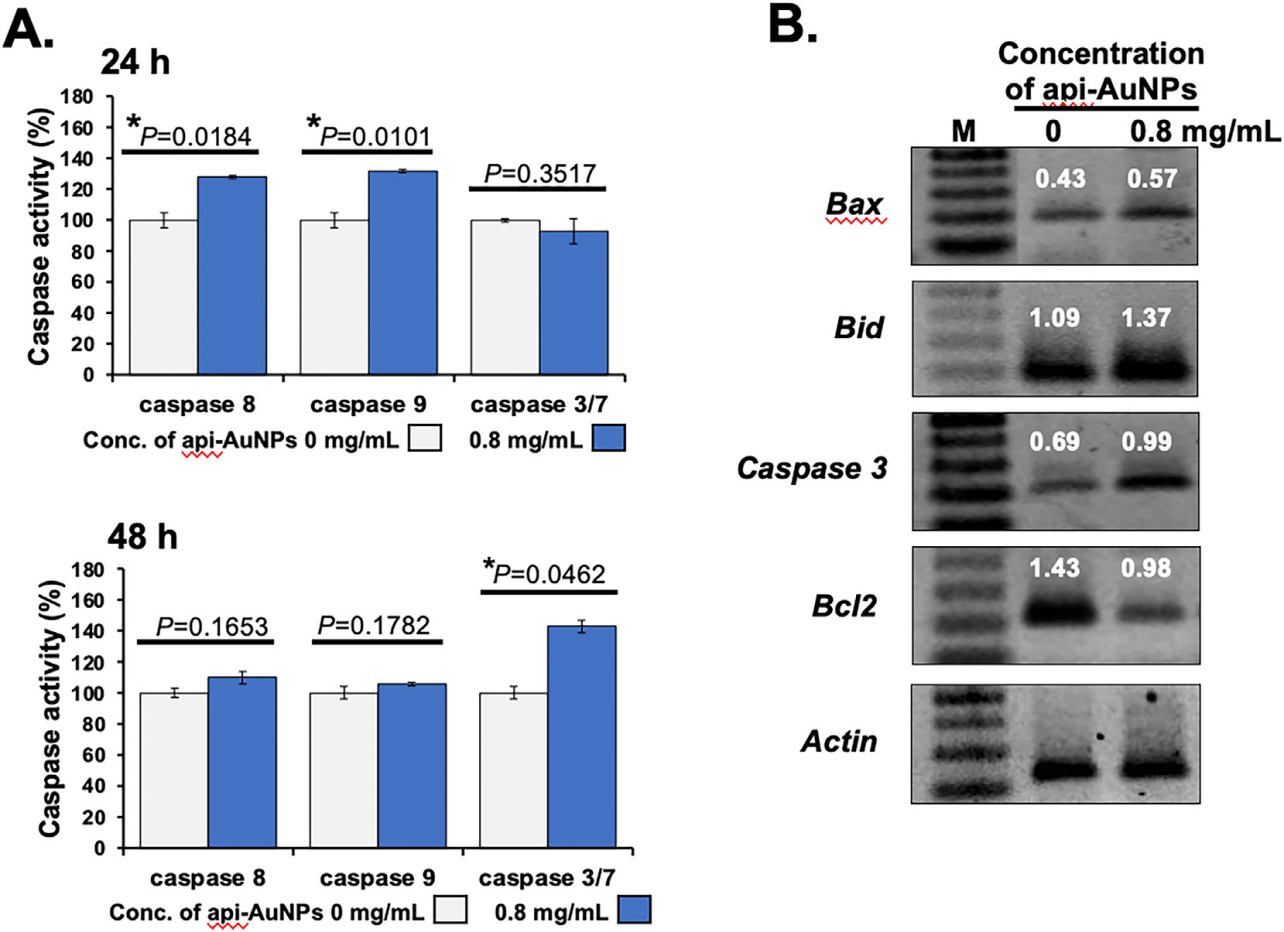
Effect of api-AuNPs on caspase activities and gene expression. (A) Activities of caspase-8, -9, and 3/7 were measured using luminescence assay. Values are presented as mean ± SD (n = 3). The ∗*P* < 0.05 is considered as statistically significant. (B) The expression of apoptotic and survival genes was determined by RT-PCR. The numbers represented the fold change from the normalized internal control (*Actin*).

Caspase -8, -9, and -3/7 are the hallmark for the investigation of apoptotic pathways [62], in which the caspase-8 activation constitutes the extrinsic pathway, while caspase-9 activation mediates the intrinsic pathway. To investigate the involvement of caspases in api-AuNP-induced apoptosis, caspase-8, -9, and -3/7 activities of KKU-M055 cells treated with api-AuNPs for 24 h and 48 h were measured using the Caspase-Glo kits. As shown in Figure 4A, the activities of caspase-8 and -9 were significantly increased after 24 h treatment when compared to the untreated cells (*P* = 0.0184 and *P* = 0.0101, respectively), and gradually decreased over the 48 h incubation period. In contrast to caspase-8 and -9, caspase-3/7 activities were significantly increased after 48 h treatment with api-AuNPs (*P* = 0.0462), but not after 24 h. This finding was in concordance with flow cytometry result that apoptotic cells were detected after 48 h. These results indicated that the api-AuNPs provoked ROS production in KKU-M055 cells that could induce apoptosis through both extrinsic and intrinsic pathways. In extrinsic apoptosis pathway, ROS activated death receptors such as FasR, TRAIL-R1/2, and TNF-R1, which recruited adaptor proteins and procaspase-8 to form complex resulting in activation of caspase-8, followed by activation of caspase-3/7 and triggering apoptosis. In intrinsic apoptosis pathway, ROS caused mitochondrial membrane opening resulting in the release of cytochrome C into the cytosol. Cytochrome C formed the apoptosome complex with procaspase-9 leading to caspase-9 activation, which then activated effector caspase-3/7 resulting in cleavage of cellular proteins and cell death by apoptosis [63]. However, the delayed increased caspase-3/7 activities suggest that the effector caspases are triggered by initiator caspases-8 and -9, and subsequently activate apoptosis [64]. Indeed, several studies have demonstrated that green synthesized AuNPs provoke apoptosis via controlling apoptotic pathways in several types of cancer cells [65, 66].

### 3.5 Effect of api-AuNPs on the expression of apoptotic and survival genes in CCA cell lines

**Figure 5:**
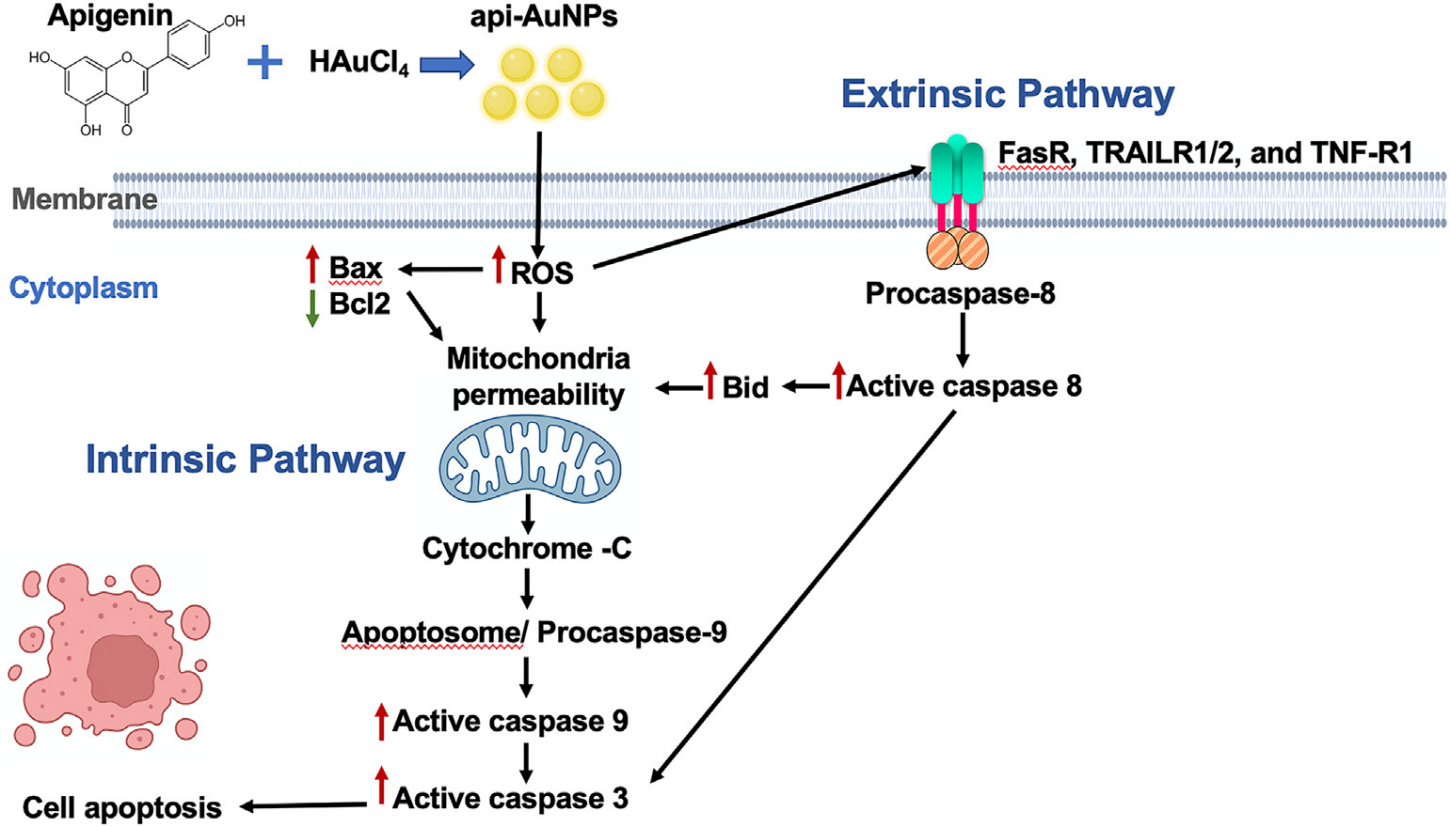
Schematic representation of api-AuNPs-mediated apoptosis in CCA cells via the activation of both intrinsic and extrinsic pathways. Intrinsic pathway involves mitochondrial mediated release of caspase-9 while extrinsic pathway involves release of caspase-8. Both caspase-8 and-9 activated effector caspase-3, and-7 which executed apoptosis.

To explore the molecular mechanisms underlying the apoptosis of KKU-M055 cells, the expression of apoptotic and pro-survival genes was analyzed after treatment with 0.8 mg/mL of api-AuNPs for 48 h. As shown in Figure 4B, the expression of *Bax*, *Bid*, and *Caspase3* was increased in api-AuNPs-treated cells compared to the untreated KKU-M055 cells. Moreover, api-AuNPs inhibited *Bcl2* expression in KKU-M055 treated cells. These data were strongly agreed with reported evidence that apigenin has the ability to block cancer growth via regulation of *Bax*, *Bcl-2*, and the caspase family in prostate, colon, lung, and breast cancer [46, 67, 68, 69, 70, 71, 72, 73]. Overall, our findings suggest that the api-AuNPs inhibit CCA cell proliferation and induce apoptosis through ROS production, which involves the activation of both intrinsic and extrinsic apoptosis pathways leading to the upregulation of apoptotic genes and suppression of pro-survival genes (Figure 5).

### 3.6 The api-AuNPs suppress the migration of CCA cells

**Figure 6:**
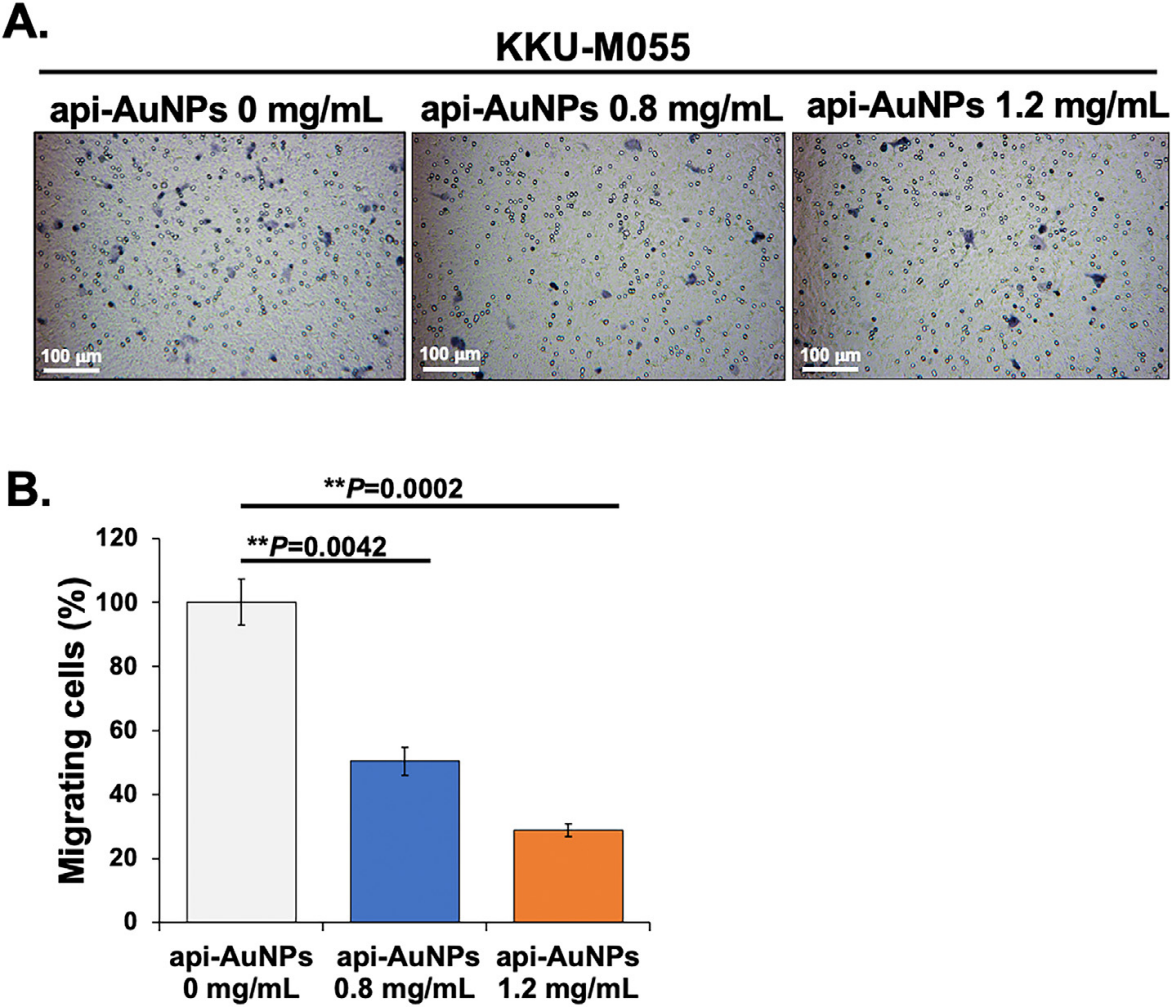
The api-AuNPs inhibit KKU-M055 migration. KKU-M055 was treated with api-AuNPs for 48 h and then cell migration was evaluated using Transwell migration assay (A) Representative results showed the effect of api-AuNPs on cell migration. (B) Quantitative data showed the significant decrease of migrating cells treated with api-AuNPs compared to untreated control. Data are shown as mean ± SD. Statistically significantly different compared to control at: ∗∗p < 0.01.

Cancer cell migration plays an important role in tumor metastasis [74]. The effect of api-AuNPs on the migration ability of KKU-M055 cells was investigated using the Transwell assay. We found that the api-AuNPs significantly suppressed migration of KKU-M055 cells (Figure 6A) 49.66 ± 4.3% and 71.14 ± 1.95% with 0.8 mg/mL and 1.2 mg/mL of api-AuNPs, respectively (Figure 6B). In line with other reports [75, 76, 77, 78], our findings indicated that api-AuNPs have potential functions in the anti-migration of multiple cancer cells including CCA.

### 3.7 The anti-angiogenic effects of api-AuNPs on endothelial cells

**Figure 7:**
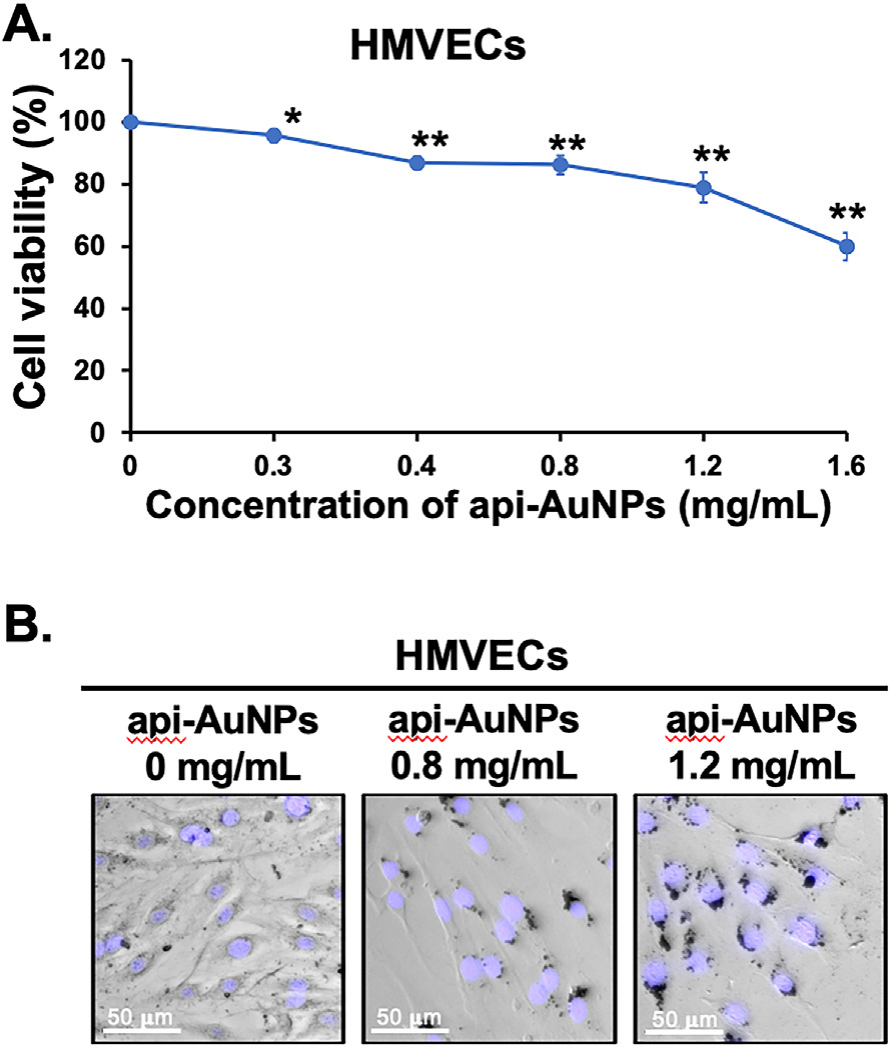
Cytotoxicity of api-AuNPs against HMVECs. (A) The number of viable cells after treatment was calculated with respect to untreated cells. The experiments were performed in triplicate and the data are expressed as mean ± SD. (B) Fluorescence micrographs using brightfield + Hoechst 33258 staining of HMVECs treated with or without api-AuNPs.

In addition to invasive cancer cells that promote tumor metastasis, tumor vascular cells that control tumor angiogenesis also collaborate with and support cancer cells to drive tumor malignant transformation. Increased tumor angiogenesis is associated with poor prognosis in CCA patients [3]. To address whether the api-AuNPs potentially inhibit tumor angiogenesis, angiogenic function including cell proliferation, migration, and tube-like formation of HMVECs were determined. The anti-proliferative effect of api-AuNPs against HMVECs was performed using the MTS assay. As shown in Figure 7A, the api-AuNPs significantly inhibited the proliferation of HMVECs in a dose-dependent manner. Moreover, morphological changes were observed when the HMVECs were treated with api-AuNPs for 48 h as compared to untreated control cells (Figure 7B).

**Figure 8:**
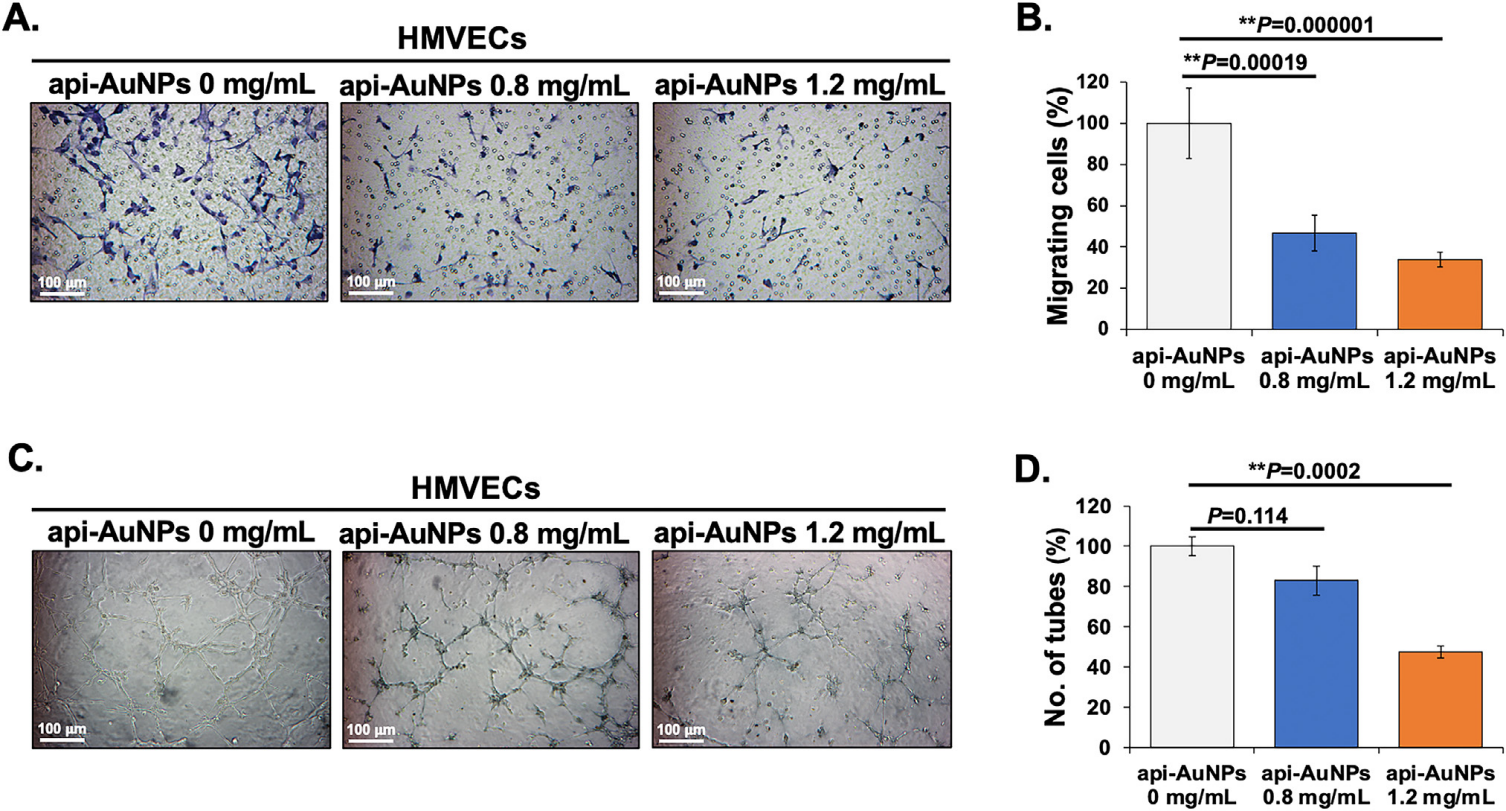
The api-AuNPs inhibited endothelial cell migration and tube-like structure formation. (A) Representative images showed the effect of api-AuNPs on the migration of HMVECs. (B) The number of HMVECs migrated through the membrane were quantified. Data are shown as mean ± SD. (C) Representative images showed the effect of api-AuNPs on tube formation of HMVECs. (D) The number of tube-like networks was quantified. Data are shown as mean ± SD. The ∗*P* < 0.05 and ∗∗*P* < 0.01 are considered as statistically significant.

We performed the migration assay of HMVECs in the presence of api-AuNPs using Transwell. The api-AuNPs significantly reduced the migration of HMVECs by 53.17 ± 8.64% and 66.14 ± 3.55% of the untreated control (Figure 8A) with 0.8 mg/mL and 1.2 mg/mL of api-AuNPs, respectively (Figure 8B).

The effect of api-AuNPs on the tube-like structure formation of HMVECs was subsequently investigated. We found that the number of tubular structures was significantly decreased by 17.04 ± 7.23% and 52.49 ± 3% (Figure 8C) with the 0.8 mg/mL and 1.2 mg/mL of api-AuNPs, respectively (Figure 8D). Several studies reported that apigenin compounds suppressed tumor angiogenesis through the inhibition of human umbilical vein endothelial cell (HUVECs) motilities and reduction of tube-like formations [79, 80, 81]. Moreover, apigenin-capped AuNPs have been reported to inhibit the growth of new blood capillaries *in vivo* in chick embryo chorioallantoic membrane (CAM) assay [37]. In contrast, Tu and colleagues showed that apigenin treatment enhanced the angiogenesis of HUVECs via caveolin-1 after hypoxia-reoxygenation [81]. Although we currently have insufficient knowledge to explain the conflict evidence, it may attribute to distinct aspects involved such as different types of endothelial cells, divergent pathogenic events, and/or individual molecular signaling mechanism. Nonetheless, our findings suggest that apigenin may inhibit tumor angiogenesis in CCA.

## 4 Conclusion

The green synthesis of apigenin conjugated gold nanoparticles was elucidated in the present study. The api-AuNPs mediate anti-proliferation of CCA cells through the activation of both intrinsic and extrinsic apoptosis pathways. They also suppress the angiogenesis process. Our findings for the first time demonstrate that the api-AuNPs exert the dual action in anti-cancer and anti-angiogenic effects. Therefore, the api-AuNPs may be a promising agent for CCA therapy. Animal models in the next step should be warranted to evaluate their efficacy outcomes.

## Declarations

### Author contribution statement

Nipaporn Ngernyuang: Performed the experiments; Analyzed and interpreted the data; Contributed reagents, materials, analysis tools or data; Wrote the paper.

Molin Wongwattanakul; Wannit Charusirisawad: Performed the experiments.

Rong Shao: Contributed reagents and materials; Wrote the paper.

Temduang Limpaiboon: Conceived and designed the experiments; Analyzed and interpreted the data; Wrote the paper.

### Funding statement

Nipaporn Ngernyuang was supported by Office of the Permanent Secretary, Ministry of Higher Education, Science, Research and Innovation [RGNS 63-126].

### Data availability statement

No data was used for the research described in the article.

### Declaration of interest's statement

The authors declare no conflict of interest.

### Additional information

No additional information is available for this paper.
